# Tracking nickel uptake pathways in hyperaccumulator plants using a ^61^Ni-enriched stable isotope tracer in soil

**DOI:** 10.1007/s00216-026-06539-6

**Published:** 2026-05-02

**Authors:** Simone Trimmel, Alexander V. Epov, Nadine Abu Zahra, Tobias Berger, Thomas Prohaska, Markus Puschenreiter, Antonia Siebenbrunner, Alice Tognacchini, Stefan Wagner, Johanna Irrgeher

**Affiliations:** 1 Montanuniversität Leoben, Department General, Analytical and Physical Chemistry, Chair of General and Analytical Chemistry, Leoben, Austria; 2 Montanuniversität Leoben, Department General, Analytical and Physical Chemistry, Chair of Physical Chemistry, Leoben, Austria; 3 BOKU University, Department of Forest- and Soil Sciences, Institute of Soil Research (IBF), Vienna, Austria

**Keywords:** Stable isotope tracing, Isotope pattern deconvolution (IPD), Metal hyperaccumulation, Bioavailability, ICP-MS, RHIZOtest

## Abstract

Understanding the mechanisms of nickel (Ni) uptake by hyperaccumulator plants is essential for advancing sustainable phytomanagement. In this study, saponite materials containing either isotopically natural or ^61^Ni-enriched Ni were synthesised and applied in RHIZOtest experiments with *Odontarrhena chalcidica*. The amendments were mixed with two ultramafic soils differing in Ni content, alongside a serpentinite control. Ni bioavailability and uptake were evaluated via elemental and isotopic analysis of plant digests and diffusive gradients in thin films (DGT). Stable isotope spiking with ^61^Ni allowed tracing of amendment-derived Ni uptake into plant tissues, even though total Ni mass fractions in planted versus unplanted soils did not indicate significant mobilisation during the 14-day growth period. Isotope pattern deconvolution (IPD) revealed clear shifts in Ni isotopic composition in both plant and DGT samples. Tracer uptake was more pronounced in the low Ni soil, with amendment-derived Ni (*x*_amendment_) contributing 19.3 ± 5.0% of total Ni in shoots, compared to 7.7 ± 1.8% in the high-Ni soil. In standard solutions containing 50 ng g^−1^ total Ni, isotope pattern shifts were still detectable at enrichment levels as low as 0.01% *x*_spike_ (≈ 5 pg g^−1 61^Ni). The findings demonstrate the sensitivity of stable isotope spiking combined with IPD in the detection of subtle uptake processes, even in short-term experiments. This approach enables the differentiation of various Ni sources in soil–plant systems that would not be achievable with quantification alone, and can thereby provide new insights into how soil mineralogy influences uptake dynamics in metal-hyperaccumulating species.

**Graphical abstract:**

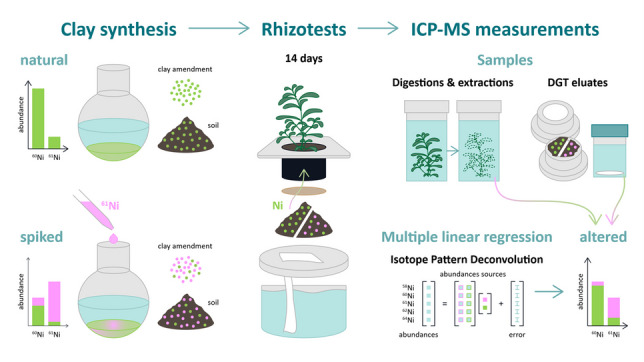

**Supplementary Information:**

The online version contains supplementary material available at 10.1007/s00216-026-06539-6.

## Introduction

Metal hyperaccumulator plants are capable of acquiring element contents in their aboveground tissues that are 100 to 1000 times higher than those found in non-accumulating species, without exhibiting symptoms of toxicity. A common threshold used to define nickel (Ni) hyperaccumulation is > 1000 µg g^−1^ Ni in shoot dry matter [1, 2]. While some physiological mechanisms underlying metal hyperaccumulation are well understood already, such as the translocation of metals from roots to shoots [3], the details of the rhizosphere processes that govern the increased mobilisation from soil are still a matter of debate [4]. Yet, deeper insights into these processes would be highly valuable to sustainable soil management strategies such as agromining [5].

Root exudates contain a mixture of organic acids and ligands such as phytometallophores which can bind metal ions in stable chelate complexes [6]. Even though their contribution to metal bioavailability is not significant under hydroponic conditions [7], experiments involving plant-soil contact suggest that they may promote solubilisation of minerals, which increases metal availability in the rhizosphere [8]. Additionally, rhizobacteria also contribute to metal hyperaccumulation by increasing water-soluble metal concentrations available to plants, likely via exuding organic ligands [9, 10].

So far, natural Ni isotope variations in plant metabolism have been explored in a few studies [11, 12]. Reported fractionation between a nutrient solution and the plant tissue is generally small, with *Δ*(^60^Ni/^58^Ni) values ranging from approximately −0.90 ‰ to −0.63 ‰ in hyperaccumulators, and around −0.21 ‰ in non-hyperaccumulating species [13]. In nature, Ni exhibits only very limited isotopic variability [14], which poses a significant challenge for source attribution based solely on natural Ni isotope ratios.

Ever since Nobel laureate George de Hevesy’s original idea of using radioactive isotopes to tag and trace sample compartments [15], they have demonstrated great potential and found manifold applications in science. Radioisotope spikes have been applied successfully to observe that hyperaccumulator plants access the same exchangeable pools as non-accumulators [16–19]. Yet, while the use of radiotracers comes with environmental and safety concerns, modern analytical techniques such as inductively coupled plasma mass spectrometry (ICP-MS) no longer need to rely on radioactivity to detect specific isotopes.

Enriched stable isotope spikes can be used to shift the isotopic composition of a material beyond natural levels, without affecting biological processes [20–24]. They have been successfully applied to investigate micronutrient uptake and transport in plants, for example, by labelling fertilisers with ^67^Zn to trace plant uptake [25]. However, enriched stable isotopes have not yet been used to investigate Ni uptake in hyperaccumulator species, nor to disentangle the contribution of mineral amendments to plant-available Ni. Thus, the potential of stable Ni isotope spiking to resolve mechanisms of hyperaccumulation remains unexplored.

Following analyte-matrix separation, the precision achievable with quadrupole ICP-MS (ICP-QMS) is fully adequate to determine this shifted isotopic composition in samples [25, 26]. The main challenge in the application of stable isotope spikes lies in determining the contributions of the individual input sources [27, 28]. Here, isotope pattern deconvolution (IPD) serves as a versatile data processing strategy to separate overlapping isotopic fingerprints within a sample.

Introduced in 2006 as a simplified alternative to double-spike isotope dilution calculations, IPD allows to identify and quantify distinct sources of an element [29]. The process involves an initial calibration step using multiple linear regression with certified reference materials (CRMs). By fitting the observed isotopic patterns to the composition stated in the certificates, the slopes can be determined. Based on these slopes and the isotopic composition of the individual sources, the contribution of each individual input source can be calculated. While no knowledge about the quantities of the respective sources is required, their isotopic composition must be thoroughly characterised. Consequently, all isotopes of the studied system need to be measured in this approach [27].

The presence of multiple mineralogical fractions in natural soils makes it difficult to attribute elemental contents to specific source phases. To selectively sample the labile fraction of metals in soil, the diffusive gradients in thin films (DGT) technique is a highly valuable tool. DGT samplers consist of a binding layer overlaid by a diffusive gel and a protective filter membrane housed in a piston sampling device [30]. Metal ions diffuse through the gel and are immobilised on the binding layer, allowing targeted sampling of kinetically labile analyte fractions, which comprise the plant-available fraction under diffusion-limited conditions [31]. The technique has been widely applied to investigate micronutrient and trace metal availability in soil [32–36]. It is particularly valuable in combination with stable isotope spiking to observe metal uptake dynamics into plants [37, 38]. A major advantage of DGT in the context of isotope ratio analysis is that the eluates are highly purified and free of most matrix components, thereby potentially eliminating the need for additional sample preparation steps prior to analysis [32, 39].

The central objective of the present study was to overcome the limitations imposed by natural Ni isotope variability in complex soils by combining enriched stable isotope spiking, IPD, and DGT-based assessment of the labile Ni fraction. Specifically, the use of synthetic ^61^Ni-enriched saponite as an isotopic tracer for Ni mobilisation from a defined clay mineral phase was evaluated. The synthesised material was applied in a RHIZOtest experiment on the model of *Odontarrhena chalcidica* (formerly *Alyssum murale*), a plant species which has already been well characterised for its Ni hyperaccumulation properties [40–44]. The experimental setup included multiple soil treatments with and without plant growth to assess the role of root-induced processes in Ni mobilisation.

## Materials and methods

Ultrapure water (18.2 MΩ cm) was produced using a Milli-Q element system (Merck Millipore, Germany). Nitric acid (HNO_3_, *w* = 65%, a.r. grade; Chem-Lab, Belgium) and hydrochloric acid (HCl, *w* = 37%, p.a. grade; Carl Roth GmbH, Germany) were subjected to further purification by sub-boiling distillation in perfluoroalkoxy alkane (PFA) polymer units (DST-1000 and DST-4000, Savillex, USA). Ultrapure hydrogen peroxide solution (H_2_O_2_, *w* = 30%; Merck KGaA, Germany) and tetrafluoroboric acid (HBF_4_, *w* = 38%; Chem-Lab EV, Belgium) were employed for sample digestion. Further information about the applied reagents is provided in Online Resource 1, section 1 - *Reagents*.

Plastic consumables were pre-treated by immersion in HNO_3_ (*w* = 3%) for a minimum of 24 h, followed by thorough rinsing with ultrapure water and drying in a laminar-flow hood. Acid cleaning, preparation of standards, and ICP-MS measurements were performed in a cleanroom environment conforming to ISO Class 8 standards or better. An XS64 Excellence analytical balance (Mettler Toledo, USA) with a readability of 0.01 g and a division of 0.0001 g was used for weighing.

Calibration standard solutions were prepared gravimetrically. For multielement analysis, two 15-point calibration series were prepared: one based on the ICP multielement standard solution VI (Merck Certipur, Germany, details given in Online Resource 1, section 2 – *MVI calibration standards*) and one based on a phosphorus (P) single-element standard solution (Alfa Aesar, USA), ranging from (nominal) 0.5 to 20,000 ng g^−1^. For Ni isotope ratio analysis, a 9-point calibration series was prepared from a Ni single-element standard solution (Alfa Aesar, USA), ranging from (nominal) 0.1 to 100 ng g^−1^. Ni isotope ratio analysis was validated using the SRM986 Isotopic Standard for Nickel (National Institute of Standards and Technology (NIST), USA). For validation, SRM1515 Apple Leaves (NIST, USA) was digested alongside plant samples.

### Experimental design using enriched ^61^Ni

The isotopic composition of the employed ^61^Ni-enriched tracer (Trace Sciences International, Canada) is provided along with the natural isotopic composition of Ni in Table 1. To determine the limit of detection and the minimum tracer enrichment detectable with reasonable analytical uncertainties, mixtures of pure natural Ni (from a single-element standard, Alfa Aesar, USA) and enriched ^61^Ni tracer were prepared, with a total Ni mass fraction of 50 ng g^−1^, and ^61^Ni molar fractions ranging from 1.14% to 91.1% (corresponding to mass fractions of ^61^Ni-enriched Ni metal powder from 0 to 100%). The analysis was conducted using a quadrupole ICP-MS instrument as specified in “ICP-MS analysis.”

**Table 1:** Abundance of individual Ni isotopes in ^61^Ni-enriched Ni metal powder (*A*_enriched_) according to the certificate provided by Trace Sciences International, compared to the representative natural Ni isotopic composition (*A*_natural_) according to the International Union of Pure and Applied Chemistry (IUPAC) [45, 46]

Isotope	^58^Ni	^60^Ni	^61^Ni	^62^Ni	^64^Ni
*A*_enriched_/%	1.6700	4.9700	91.1000 ± 0.5000	2.1900	0.1700
*A*_natural_/%	68.0769 ∓ 0.0190	26.2231 ∓ 0.0150	1.1399 ∓ 0.0013	3.6345 ∓ 0.0040	0.9256 ∓ 0.0019

This information was a critical prerequisite for designing the subsequent plant experiment, ensuring that tracer additions would be analytically resolvable. The minimum required tracer content in the soil for the pot experiments was determined based on the limit of detection (*x*_D_) for ^61^Ni analysis and the expected uncertainties of Ni isotope ratio measurements. Based on the outcome of this preliminary experiment (see “Evaluation of ^61^Ni spike levels required for tracing”), 100 mg of ^61^Ni-enriched powder was combined with 1.5906 g of natural Ni powder, resulting in a ^61^Ni-spiked saponite blend with a theoretical molar fraction of *x*_spike_ = 5.92%.

### Experimental soils and natural serpentinite

The experimental soils used in this study were obtained from an ultramafic forest area near Redlschlag (Burgenland, Austria) which has been previously described in literature [8, 40, 41, 47, 48]. Ultramafic soil originates from the weathering of ultramafic rocks, such as serpentinite, peridotite, and dunite. These rocks are rich in iron (Fe) and magnesium (Mg), but have low levels of essential plant nutrients such as calcium (Ca), potassium (K), and phosphorus (P) when compared to common agricultural soils. In addition, they often exhibit elevated contents of potentially phytotoxic metals such as Ni, chromium (Cr), and cobalt (Co), which results in a highly endemic plant community adapted to these conditions [49–51].

Two sites with the following coordinates (given in WGS84) were sampled: 47.440528° (N), 16.317750° (E) (soil S1) and 47.439778° (N), 16.313778° (E) (soil S6). The two soils were both rich in serpentinite and were collected along a natural ultramafic gradient. Therefore, they are characterised by less (S1) or more (S6) pronounced ultramafic characteristics: pseudo-total (aqua regia extractable) Ni was 552 ± 52 mg kg^−1^ for S1 and 1465 ± 58 mg kg^−1^ for S6, while diethylene triamine pentaacetic acid (DTPA) extractable Ni was 41.6 ± 0.5 mg kg^−1^ for S1 and 158 ± 7 mg kg^−1^ for S6. A detailed characterisation of the two experimental soils can be found in a previous study [40]; a summary is provided in Online Resource 2, Table A1.

As part of the experimental design, a naturally occurring serpentinite rock was used alongside the two synthetic materials to assess the influence of different sources on Ni uptake. The serpentinite material was collected manually from the surface of a quarry near Pilgersdorf (Burgenland, Austria, 47.438722° (N), 16.314528° (E), WGS84) within a serpentinised ultramafic body. Qualitative and semi-quantitative analyses of total mineral contents were performed at the Institute of Applied Geology (BOKU University, Vienna, Austria). Details are given in Online Resource 1, section 3 – *Total mineral analysis of natural serpentinite*. Briefly, the sampled material consists of about *w* = 85% serpentine lizardite, a silicate mineral from the serpentinite group. Other components are chlorite (*w* = 13%), magnetite (*w* = 1–2%), and magnesite (traces).

### Preparation and characterisation of clay amendments

The synthesis of the saponite soil amendments (both ^61^Ni-spiked and non-spiked) was modified based on an existing protocol for the synthesis of Mg-rich saponite [52]. Preliminary trials were conducted to ensure controlled and reproducible incorporation of Ni into the clay structure. Starting from a Ni:Mg ratio of 1:75 and increasing gradually to 50:50, the potential of partial substitution of Mg^2+^ by Ni^2+^ in the octahedral sheets was explored. In parallel, synthesis parameters such as duration, temperature, and stirring conditions were adjusted to improve the crystallinity and reduce the fraction of readily soluble Ni. The final synthesis procedure was designed to achieve a Ni content of *w* = 0.5% in the saponite and is described in detail along with the subsequent washing procedure in Online Resource 1, section 4 – *Saponite synthesis*.

After air-drying, three replicates of both synthetic saponite materials and the natural serpentinite were digested for characterisation. For this, 0.1 g per replicate was weighed into perfluoroalkoxy (PFA) vessels and heated to 110 °C on a hotplate for 4 h along with 7 mL HNO_3_ (*w* = 65%) and 1 mL HBF_4_ (*w* = 38%), followed by evaporation to near-dryness. In the next step, 4 mL HCl (*w* = 32%) and 2 mL HNO_3_ (*w* = 65%) were added. After another period of heating at 110 °C for 4 h, the samples were again evaporated to near-dryness. Subsequently, the digests were washed with 0.5 mL HNO_3_ (*w* = 65%) three times, each time followed by evaporation to near-dryness. Finally, they were filled to 20 mL with HNO_3_ (*w* = 2%) and filtrated to remove residues of particles (supposedly tyre wear) in the natural serpentinite.

Additionally, to assess remaining extractable Ni contents, extraction with EDTA (*c* = 0.05 mol L^−1^) was performed for two replicates of the washed and air-dried synthetic materials and the natural serpentinite. 0.25 g to 0.5 g per replicate were mixed with 5 mL EDTA (*c* = 0.05 mol L^−1^), shaken at 20 rpm for 1 h, and filtrated through syringe filters (0.45 µm pore size).

For crystallinity assessment, the samples were investigated via powder X-ray diffraction (XRD) measurements using a BRUKER-AXS D8 Advance ECO diffractometer (Bruker Corporation, Billerica, USA) operating with a Cu Kα radiation source under 40 kV and 25 mA (*λ* = 1.5406 Å). The measurements were conducted at room temperature using Bragg–Brentano geometry and a scanning rate of 0.02 s^−1^ with an acquisition time of 3 s per step.

### Plant and soil samples

#### RHIZOtest

Seeds of *O. chalcidica* were surface-sterilised by (i) gently shaking for 5 min in ethanol (*w* = 70%) and rinsing three times in sterile ultrapure water, followed by (ii) gently shaking for 30 min in sodium hypochlorite solution (NaOCl, *w* = 2%, DanKlorix, Germany) and rinsing five times in sterile ultrapure water.

Germination of *O. chalcidica* was initiated in a hydroponic RHIZOtest system [53] which separated plant roots from the nutrient solution through a fine polyamide mesh (30 µm diameter, Sefar AG, Heiden, Switzerland) at the bottom of each cylinder (40 mm diameter). A total of 3000 surface-sterilised seeds were germinated in 72 cylinders, placed in floating platforms inserted into 6-L buckets containing a germination solution of CaCl_2_ (0.6 mmol L^−1^) and H_3_BO_3_ (2 µmol L^−1^). Growth cabinet (SE59-AR2cLED, CLF Plant Climatics, Germany) conditions were set to a day/night temperature of 24 °C/18 °C and a humidity of 60%.

After a 3-week germination phase, one seedling per cylinder was retained, resulting in a total of 43 surviving seedlings. These were grown for an additional 5 weeks to develop a root mat, with a nutrient solution provided in the buckets. The nutrient solution contained H_3_BO_3_ (*c* = 10 µmol L^−1^), KH_2_PO_4_ (*c* = 0.1 mmol L^−1^), Ca(NO_3_)_2_ (*c* = 0.5 mmol L^−1^), MgSO_4_ (*c* = 1 mmol L^−1^), CuSO_4_ (*c* = 0.2 µmol L^−1^), MnCl_2_ (*c* = 1 µmol L^−1^), ZnSO_4_ (*c* = 2 µmol L^−1^), Na_2_MoO_4_ (*c* = 0.2 µmol L^−1^), NaFe(III)EDTA (*c* = 5 µmol L^−1^), and KNO_3_ (*c* = 1 mmol L^−1^). Ten samples of *O. chalcidica* were harvested to monitor the elemental contents and Ni isotopic composition at the startpoint before the soil contact phase.

**Fig. 1:**
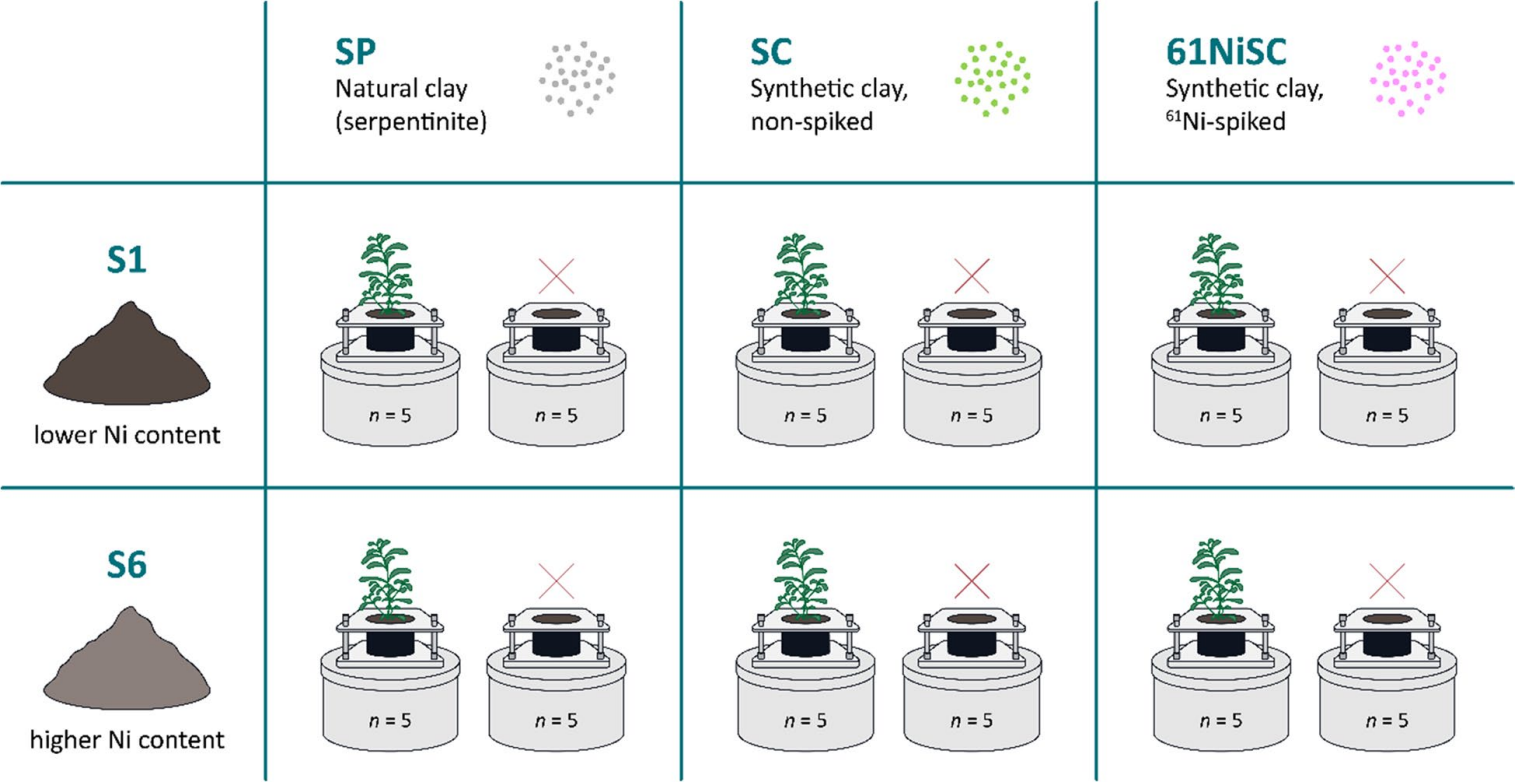
Overview of the RHIZOtest experimental setup

For the soil contact phase, portions of the two ultramafic soils S1 (lower Ni content) and S6 (higher Ni content) were each amended with one of the three materials: (1) natural ground serpentinite, (2) non-spiked saponite, or (3) ^61^Ni-spiked saponite. A visual representation of the setup is provided in Fig. 1.

For each treatment, approximately 2 g of soil was mixed with 0.3 g of the respective amendment within each RHIZOtest unit. The amendment rate was selected for analytical purposes: Based on pseudo-total Ni contents, this corresponds to a theoretical *x*_spike_ of 0.66% for S1 and 0.27% for S6, which enables clear isotopic differentiation between spiked and non-spiked systems. The lower compartment of the RHIZOtest units was filled with a soil contact solution containing Ca(NO_3_)_2_ (*c* = 0.25 mmol L^−1^), MgSO_4_ (*c* = 0.5 mmol L^−1^), KNO_3_ (*c* = 0.5 mmol L^−1^), and KH_2_PO_4_ (*c* = 0.05 mmol L^−1^).

Root-induced changes in the rhizosphere were assessed by comparing soil from planted RHIZOtest devices (rhizosphere soil) to unplanted controls (bulk soil) in identical setups. Both planted and unplanted devices were replicated five times. The unplanted devices were covered with a lid for the duration of the soil contact phase. The buckets were placed in the growth cabinet with the same conditions as during the germination phase, adding a 16-h photoperiod with a light intensity of 400 μmol photons m^−1^ s^−1^.

After 14 days, the shoots were cut as close to the bottom as possible and put into paper bags for transport. The roots were discarded. The moist soil was scraped off using plastic spatulas and transferred into polypropylene (PP) tubes. The plants were oven-dried at 60 °C for 4 days. After determining the dry biomass, microwave-assisted digestion was performed based on a procedure established in an earlier study [54] using 5 mL of HNO_3_, 1 mL of H_2_O_2,_ and 0.1 mL of HBF_4_. The samples were heated to 200 °C for 15 min in a Multiwave Pro microwave oven (Anton Paar, Austria) along with three replicates of SRM1515 Apple Leaves and three procedural blanks.

#### DGT sampling

DGT binding layers made from agarose cross-linked polyacrylamide (APA) gel containing Chelex-100 resin (sodium form, particle size 37–75 µm) as cation binding phase were prepared according to literature [30]. DGT devices were assembled by stacking DGT base, Chelex-100 binding layer (thickness 0.4 mm), APA diffusive gel (thickness 0.8 mm), and polyethersulfone (PES) filter membrane (thickness 0.14 mm, pore size 0.45 µm) on top of each other, and firmly closed with the DGT cap. A ~ 2-mm-thick layer of the moist soil samples (corresponding to ~ 2 g of soil dry weight) was placed onto the exposure window of the DGT devices 1 day after plant harvest and soil collection from the RHIZOtest experiment. DGT sampling was conducted for 24 h, noting the times of soil deployment and removal. The exact deployment times are given in Online Resource 2, Table A3. Six procedural blanks were processed along with the samples.

After disassembling the DGT devices, the binding layers were rinsed with ultrapure water. Bound analytes were recovered by shaking in 5 mL HNO_3_ (*c* = 1 mol L^−1^) at 180 rpm for 24 h. An eluent volume of 5 mL was chosen in this study to maximise the elution efficiency for Ni in order to minimise the risk of isotopic fractionation. It was shown in literature that a Ni elution efficiency of 98 ± 2% can be achieved with 5 mL, with no significant improvement when the eluent volume is increased to 10 mL [55]. Therefore, 5 mL was deemed as the optimal volume to achieve the highest possible Ni concentrations without compromising the recovery.

### ICP-MS analysis

Sixteen elements (Li, Be, Na, Mg, Al, P, K, Ca, Mn, Fe, Co, Ni, Cu, Zn, Cd, and Pb) were quantified in plant digests, DGT eluates, as well as both acid digests (total contents) and EDTA extracts of the amendments. The primary motivation for this analysis was to identify elemental contents that might interfere with Ni isotope ratio measurements—particularly Fe, Zn, and Cu, which may cause isobaric or polyatomic interferences on *m*/*z* 58, 60, and 61, or Ca, which is associated with matrix effects especially in the plant samples.

For multielement analysis, plant digests (*n* = 40) were diluted by a factor of 10 with ultrapure water, clay digests (*n* = 9) were diluted by a factor of 10 with HNO_3_ (*w* = 2%), and DGT eluates (*n* = 60) were diluted by a factor of 3.125 with ultrapure water. Dilution factors were selected based on the expected Ni content and matrix composition of the respective sample types to ensure signals within the optimal working range while avoiding excessive matrix load. For Ni isotope ratio analysis, the samples were diluted to achieve 1–20 ng g^−1^ Ni and 2% HNO_3_. This range of mass fractions ensured that all Ni isotopes were measured in the same detector mode (pulse mode), which is a prerequisite for obtaining comparable signals for the calculation of isotope ratios.

Multielement analyses were performed using a NexION 5000 ICP-MS/MS instrument, and Ni isotope ratio analyses were conducted using a NexION 2000 ICP-MS instrument (both PerkinElmer, USA). Possible carry-over effects were monitored by measuring analysis blanks of HNO_3_ (*w* = 2%) after every four samples. After every ten samples, quality control (QC) solutions were measured. To monitor instrumental drift and correct for matrix effects, indium (In) was used as an internal normalisation standard. After each sample, a rinse with HNO_3_ (*w* = 3%) was performed. Details about the instrumental parameters are given in Online Resource 1, section 4 – *Multielement analysis (NexION 5000)* and section 5 – *Ni isotope ratio analysis (NexION 2000)*.

### Data processing

The acquired ICP-MS data was processed using the Syngistix software version 2.5 (PerkinElmer, USA). For data evaluation and statistical testing, Microsoft 365 Excel version 2211 (Microsoft, USA) was used. In all statistical tests, *p*-values below 0.05 were regarded as significant. Limits of detection (*w*_D_) and quantification (*w*_Q_) were calculated based on the standard deviation (*s*) of the procedural blanks (mean + 3*s* and mean + 10*s*).

Ni isotope ratios given in Online Resource 2 are reported using the delta (*δ*) notation (in ‰), where the isotope ratio is expressed relative to an isotopic reference standard (NIST SRM986) according to Eq. 1.

1 $${{\delta }_{\mathrm{standard}}({}^{61}\mathrm{Ni}/{}^{62}\mathrm{Ni})}=\frac{{R}_{\mathrm{sample},\text{ corrected}}}{{R}_{\mathrm{standard},\text{ mean}}}-1$$

*R*_sample, corrected_: isotope ratio of sample after blank and IIF correction

*R*_standard, mean_: isotope ratio of reference standard measured before and after sample

#### DGT data processing

The concentration of Ni at the interface between soil and DGT sampler (*c*_DGT_) was calculated using Eq. 2.

2 $${c}_{\mathrm{DGT}}=\frac{M\Delta g}{{{D}^{\mathrm{mdl}}A}_{\mathrm{p}}t}$$

Δ*g*: thickness of material diffusion layer

*D*^mdl^: diffusion coefficient of analyte in material diffusion layer

*A*_p_: geometric area of the DGT device window (this study: 3.14 cm^2^)

*t*: deployment time (see Online Resource 2, Table A3)

Thereby, Δ*g* is the sum of the thickness of the diffusive gel layer (this study, *δ*^g^ = 0.080 cm) and the thickness of the membrane filter (this study, *δ*^f^ = 0.014 cm), resulting in Δ*g* = 0.094 cm. Further details on DGT data processing are given in Online Resource 1, section 6 – *DGT data processing*.

#### Isotope pattern deconvolution (IPD)

To determine the fraction of Ni originating from the ^61^Ni-enriched amendment in each sample, an IPD approach was applied based on previous studies [21, 22, 27, 29, 56]. The number of isotopes in a system *n* allows for the mathematically robust separation of *n* – 1 sources by solving a system of linear equations.

Instrumental drift was corrected from repeated measurements of SRM986. For plant samples, prior to IPD, the isotope signals were corrected for potential interferences. The correction factors were determined empirically from unspiked samples with natural Ni isotopic composition. Factors were adjusted such that the corrected signals reproduced the expected natural Ni isotope ratios within analytical uncertainty. Once established, the same correction factors were applied consistently to all plant samples, including tracer-amended ones. This empirical correction approach accounts for instrument- and matrix-specific formation rates of polyatomic species.

The validity of the correction approach was evaluated using unspiked samples, including NIST SRM 1515 and startpoint samples, for which natural Ni isotope ratios are expected. Without correction, deviations from the natural isotopic composition were observed, particularly in samples with low Ni mass fractions and, in comparison, high matrix element contents. After correction, the expected natural isotope ratios were reproduced within measurement uncertainty. The extent of the corrections depended on the sample matrix. For most plant samples, corrections were minor (typically within a few percent of the measured signal), whereas larger corrections (> 10%) were required for samples with low Ni-to-matrix ratios, such as SRM 1515 and some startpoint samples. For samples other than plants, correction for these interferences was not necessary due to the purer matrix.

Isotope abundances were then calculated from the corrected isotope ratios following Eq. 3.

3 $${A}_{\mathrm{sample}}^{i}=\frac{{R}_{i}}{{\sum }_{i=1}^{n}{R}_{i}}$$

${A}_{\mathrm{sample}}^{i}$: abundance of a single isotope in the sample

*R*_i_: isotope ratio

The molar fractions of the individual contributors to the isotopic composition in the mixture can be calculated by deconvolution of the measured isotope abundances and those from the individual input sources according to Eq. 4. In the case of Ni with five naturally occurring isotopes, *m*/*z* 58, 60, 61, 62, and 64 are considered. Due to elevated background and correspondingly high uncertainty on *m*/*z* 64, the signal of ⁶^4^Ni was not used directly but instead calculated based on the corrected isotope pattern and natural isotopic abundances.

4 $$\left[\begin{array}{c}\begin{array}{c}{A}_{\mathrm{sample}}^{58}\\ {A}_{\mathrm{sample}}^{60}\\ {A}_{\mathrm{sample}}^{61}\end{array}\\ {A}_{\mathrm{sample}}^{62}\\ {A}_{\mathrm{sample}}^{64}\end{array}\right]=\left[\begin{array}{cc}{A}_{\mathrm{natural}}^{58}& {A}_{\mathrm{spike}}^{58}\\ {A}_{\mathrm{natural}}^{60}& {A}_{\mathrm{spike}}^{60}\\ \begin{array}{c}{A}_{\mathrm{natural}}^{61}\\ {A}_{\mathrm{natural}}^{62}\\ {A}_{\mathrm{natural}}^{64}\end{array}& \begin{array}{c}{A}_{\mathrm{spike}}^{61}\\ {A}_{\mathrm{spike}}^{62}\\ {A}_{\mathrm{spike}}^{64}\end{array}\end{array}\right]\left[\begin{array}{c}{x}_{\mathrm{natural}}\\ {x}_{\mathrm{spike}}\end{array}\right]+\left[\begin{array}{c}\begin{array}{c}\begin{array}{c}{e}^{58}\\ {e}^{60}\end{array}\\ {e}^{61}\end{array}\\ {e}^{62}\\ {e}^{64}\end{array}\right]$$

*x*: molar fraction in the source

*e*: error of a single isotope

*x*_spike_ was calculated iteratively with the Microsoft Excel add-in program Solver to obtain the minimum of the weighted mean square difference between theoretical and measured isotope abundance. For each sample, the spike molar fraction (*x*_spike_) was initially set to 0, with the natural molar fraction (*x*_natural_) set to 1 – *x*_spike_. The isotopic composition of the spike was taken from the certificate of the ^61^Ni-enriched Ni powder, while the natural Ni signature was defined as the average composition across all unspiked samples of one type. Based on the set molar fractions and the known isotopic compositions of spike and natural Ni, theoretical isotopic abundances were calculated. Weights were applied to emphasise more reliable and discriminative isotopes: ^61^Ni (× 10), ^58^Ni and ^60^Ni (× 1), ^62^Ni (× 0.5), and ^64^Ni (× 0.2). The weighting scheme was based on isotopic abundance, analytical robustness, and information content: ^61^Ni was upweighted due to its role as the tracer isotope, whereas ^62^Ni and especially ^64^Ni were downweighted because of their lower abundance and higher susceptibility to interferences. The Solver was constrained to keep *x*_spike_ within the range from 0 to 1.

## Results and discussion

The complete data set is given in Online Resource 2. Table A3 presents an overview of the RHIZOtest treatments, including plant biomass data and *c*_DGT_ values. Elemental mass fractions are given in Table B1 with standard deviations of instrumental replicates (*n* = 6) in Table B2. *w*_D_ and *w*_Q_ are given in Table C1 and biases obtained on the certified values of SRM1515 in Table C2. Ni isotope ratios and obtained molar fractions are shown in Table D1 with relative standard deviations of instrumental replicates (*n* = 900) in Table D2. Table E1 and Table E2 provide the Ni isotope ratios determined in SRM1515 and SRM986.

### Evaluation of ^61^Ni spike levels required for tracing

As shown in Table 2, a proportional increase in the *δ*(^61^Ni/^60^Ni) values with the spike molar fraction (*x*_spike_) was observed in the series of mixtures of ^61^Ni-enriched and natural Ni solutions. Even with a very low *x*_spike_ of 0.01%, a shift by greater than 5 ‰ (*δ*(^61^Ni/^60^Ni) = 5.136 ‰ ± 0.028 ‰) was measured. Given that the difference in *δ*(^61^Ni/^60^Ni) between this solution and the Ni solution of natural isotopic composition (*x*_spike_ = 0) is almost 200 times higher than the combined uncertainty of the result (*U* (*δ*(^61^Ni/^62^Ni)) = 0.028 ‰), the signal can be unambiguously attributed to the spike. Relative to the total Ni mass fraction of 50 ng g^−1^, this means that already a mass contribution of 5 pg g^−1^ of ^61^Ni leads to a shift in the *δ*(^61^Ni/^62^Ni) value by approximately 5 ‰.

The relative standard deviations (RSD) of the measured ratios are between 0.49% and 2.13%, which is reported in absolute values in Table 2. At a spike level of 1.50%, the *δ*(^61^Ni/^62^Ni) values reach the target range of 500–600 ‰. Thus, even accounting for substantial measurement uncertainty due to instrumental RSD or low Ni signal in the sample solution, this approach represents a conservative and robust strategy for tracer-based experiments. To achieve a sufficiently distinct Ni isotopic composition in the synthetic saponite, the synthesis was targeted to yield a material with a *δ*(^61^Ni/^60^Ni)_SRM986_ value of approximately 800–900 ‰.

**Table 2:** *δ*(^61^Ni/^62^Ni) values obtained in a series of ^61^Ni-enriched/natural Ni mixtures in various ratios. *U *(*δ*(^61^Ni/^62^Ni)) is the standard deviation of 900 instrumental replicates

*x*_spike_/%	*δ*(^61^Ni/^62^Ni)/‰	*δ*(^61^Ni/^62^Ni)/‰	*U *(*δ*(^61^Ni/^62^Ni))/‰
	Theoretical	Measured	
0.00	0.00	−0.1514	0.0008
0.01	7.87	5.136	0.028
0.02	15.6	19.23	0.20
0.05	38.2	35.28	0.21
0.10	73.5	63.96	0.50
0.15	106	96.63	0.43
1.50	545	521.0	11.0
2.25	643	619.0	3.3
20.0	945	939.8	5.1
50.0	980	978.9	7.3
90.0	991	991.1	5.1
100	992	992.6	8.1

### Soil amendments: characterisation and impact on plant biomass

The mineralogical and elemental composition of the soil amendments (the two synthetic saponite materials and the natural serpentinite) was characterised prior to their application in the RHIZOtests. This included XRD analysis, multielement analysis and Ni isotope ratio measurements of both total digests and EDTA extracts.

**Fig. 2:**
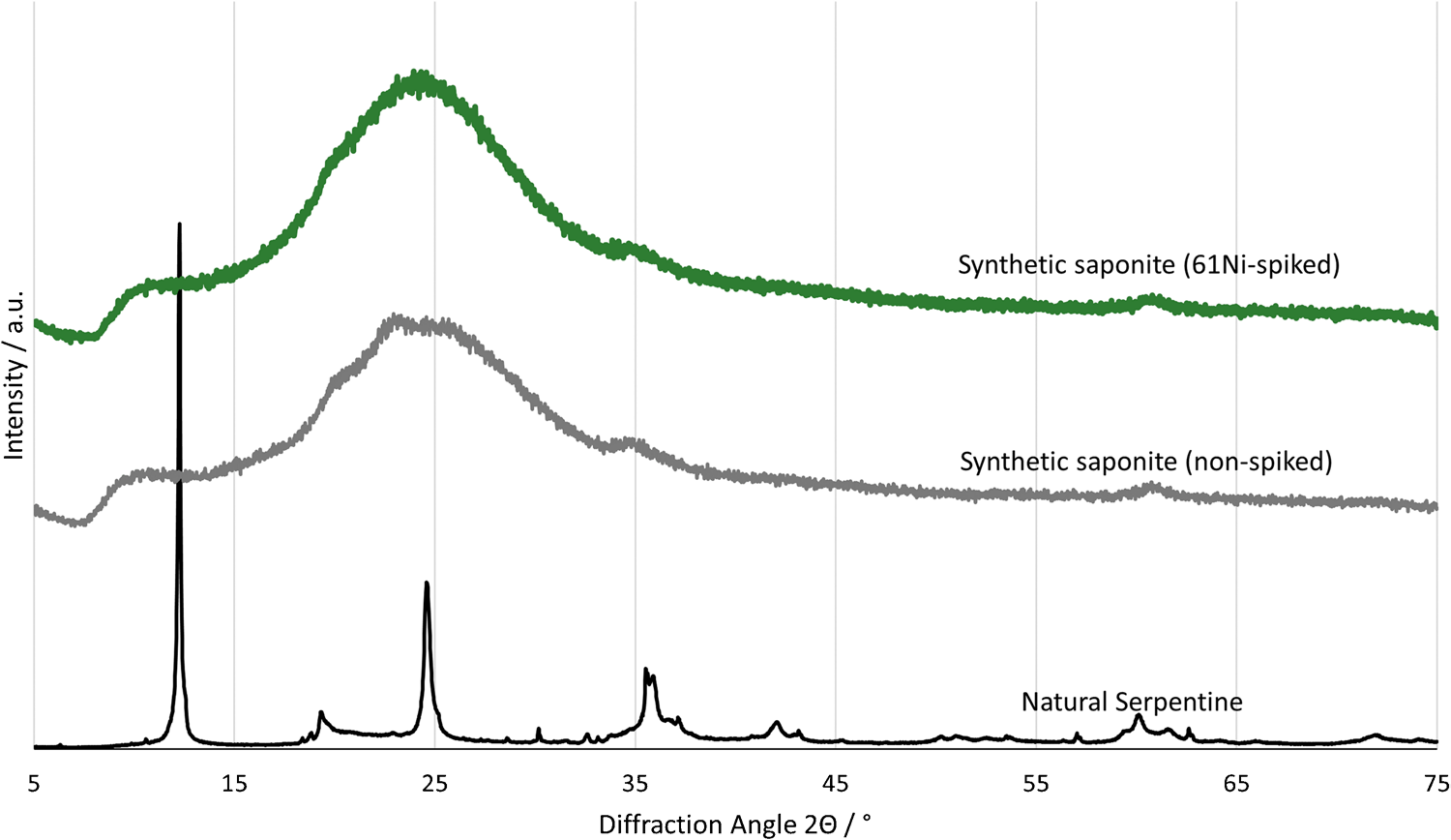
XRD plots of the three amendments: natural serpentinite (bottom line), non-spiked synthetic saponite (middle), and ^61^Ni-spiked synthetic saponite (top)

Figure 2 shows the XRD plots of the three amendments. The natural serpentinite exhibited sharp and well-defined diffraction peaks, which is indicative of a highly crystalline mineral component [57]. At the same time, the elevated background signal in the regions between 17–25° and 34–45° 2Θ suggests the presence of an amorphous phase [58]. This is in alignment with the findings of the mineralogical characterisation described in section ”Experimental soils and natural serpentinite" (and detailed in Online Resource 1, section 3 – *Total mineral analysis of natural serpentinite*), which identified serpentine, chlorite, magnetite, and magnesite as the dominant crystalline phases. The elevated background in the XRD pattern indicates that the natural amendment contains a mixture of crystalline and amorphous material. In contrast, both synthetic saponites (non-spiked and ^61^Ni-spiked) showed broader, less intense features, consistent with a lower degree of crystallinity.

As detailed in “Evaluation of 61Ni spike levels required for tracing”, the theoretical *x*_spike_ in the synthesised ^61^Ni-spiked saponite was 5.92%. The values determined by IPD in total digests and EDTA extracts were lower, at 4.54 ± 0.01% and 4.71 ± 0.04%, respectively. This difference likely reflects the actual incorporation efficiency of Ni during synthesis, as well as minor contributions from synthesis reagents or small systematic errors in isotope ratio measurements.

**Fig. 3:**
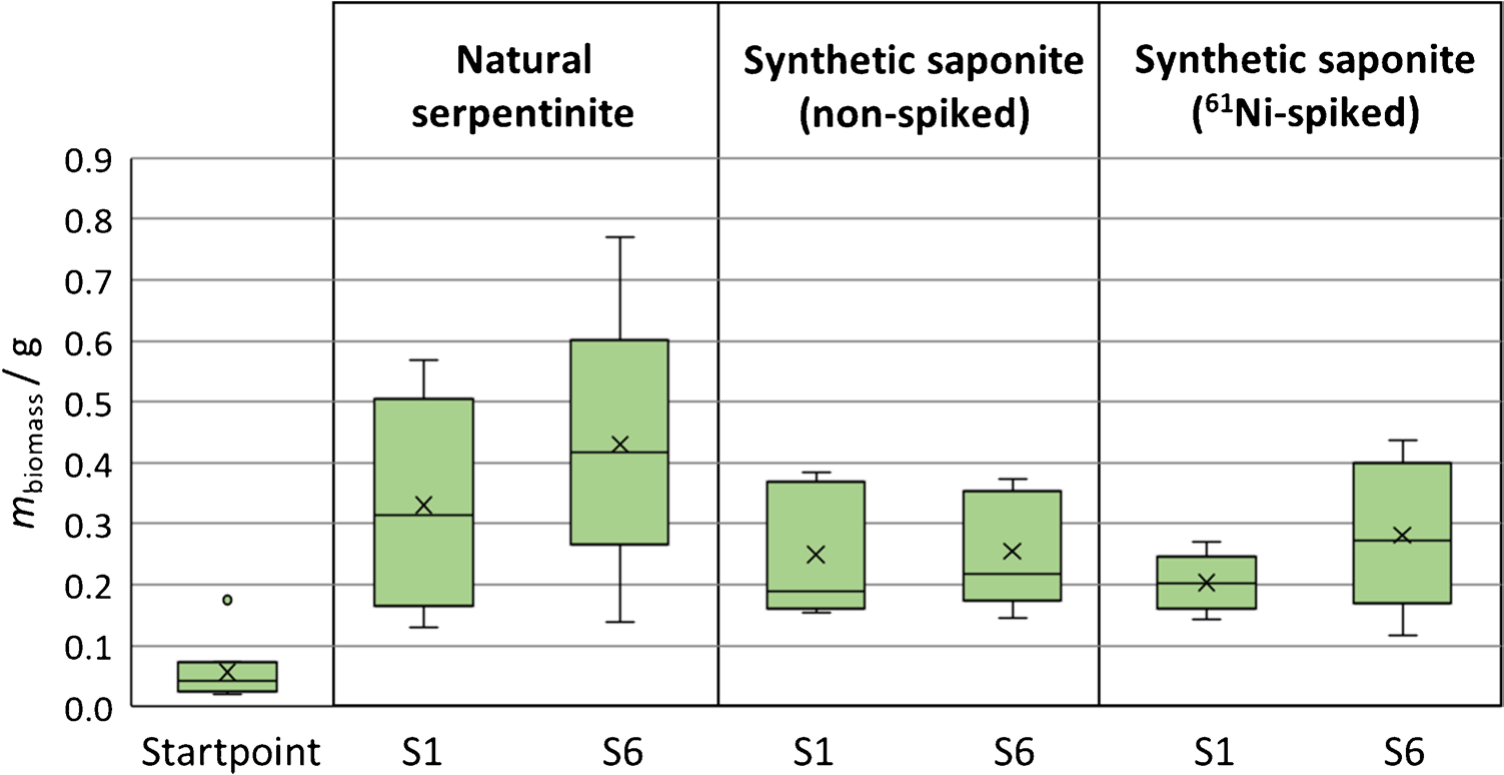
Dry biomasses of the harvested plant samples (startpoint, *n* = 10, otherwise *n* = 5 per group)

The variation of the plant biomasses across the different treatments is shown in Fig. 3. One-way ANOVAs conducted separately for each soil (excluding startpoint samples) revealed no significant differences between treatments (*p* = 0.296 for S1 and *p* = 0.207 for S6), indicating that the synthetic saponite had no measurable impact on plant growth under the experimental conditions. Negative correlations between biomasses and elemental mass fractions in the plant tissues point towards a dilution effect [59] and are detailed in Online Resource 1, section 7 – *Plant biomass*.

### Ni mobilisation from amended soils

**Fig. 4:**
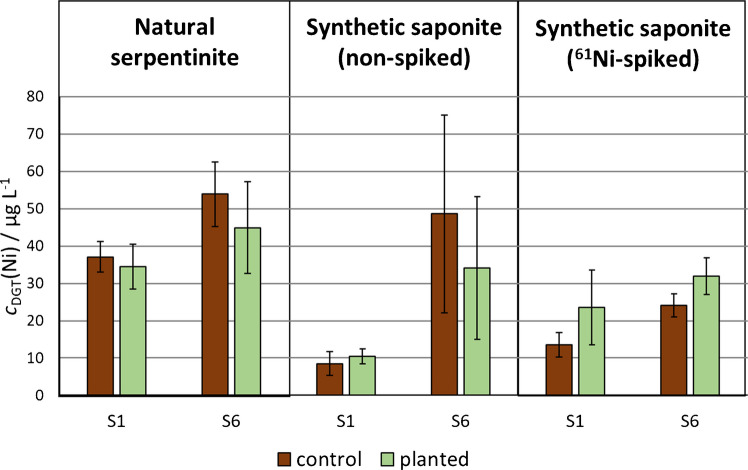
*c*_DGT_(Ni) in RHIZOtest soils after the 14-day growth period without (control) or with (planted) *O. chalcidica*. Error bars indicate standard deviations between the replicates (*n* = 5)

The DGT technique enables the assessment of the kinetically labile Ni fraction in soil, consisting of free Ni^2+^ ions in soil solution, weakly complexed Ni species that dissociate within the diffusive gel, and Ni resupplied from exchangeable solid-phase pools during deployment (for details, see Online Resource 1, section 6 – *DGT data processing*). Thereby, DGT concentrations (*c*_DGT_) reflect the Ni fraction that is dynamically available in the soil porewater and capable of diffusive transport towards a sink, approximating the pool potentially accessible for plant uptake in the rhizosphere. The *c*_DGT_(Ni) values obtained in both soils fall within the range reported for Ni-rich ultramafic soils, which typically spans from several tens to hundreds of µg L^−1^ [60, 61]. As shown in Fig. 4, no significant differences in *c*_DGT_(Ni) values were observed between planted and unplanted soils across all amendment groups. This suggests that root-induced Ni mobilisation over the 14-day growth period did not significantly affect the DGT-labile Ni pool within the amended soils.

**Fig. 5:**
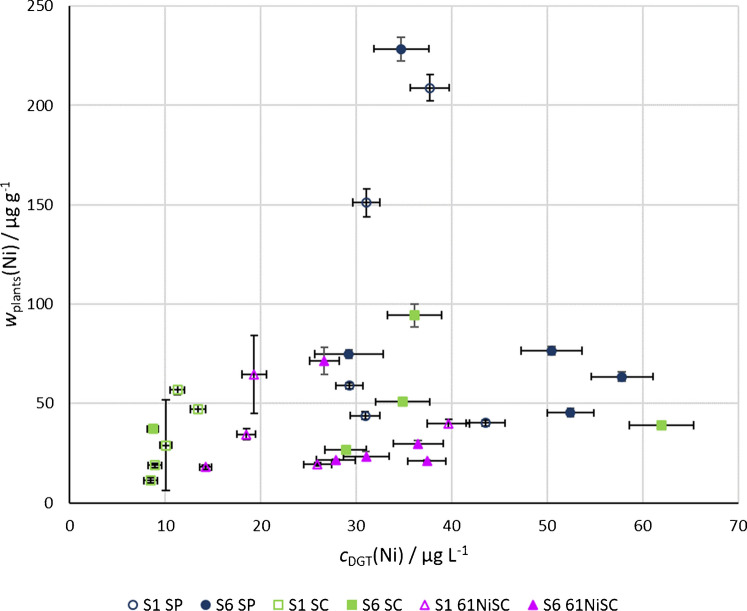
Mass fractions of Ni in plants versus *c*_DGT_ (Ni) in the corresponding amended soils. SP, serpentinite; SC, synthetic smectite; 61NiSC, ^61^Ni-enriched synthetic smectite. Error bars indicate the standard deviation of instrumental replicates (*n* = 6)

This is further underlined by the absence of significant correlations between the mass fractions of Ni in the plants and the *c*_DGT_(Ni) values in the respective amended soils, as shown in Fig. 5. Pearson correlation coefficients were calculated and tested for significance both across all samples and for each individual group of amended soils, with none of them being significant.

The 14-day growth period may have been too short for rhizosphere processes such as the exudation of chelating compounds or root-induced pH changes to significantly alter the composition of the labile Ni pool. Previous studies have reported increased labile Ni contents in the rhizosphere of Ni hyperaccumulators, suggesting root-induced dissolution of Ni-bearing minerals [8, 61, 62]. However, other authors argue that hyperaccumulators may primarily rely on efficient uptake from already labile Ni pools rather than accessing previously unavailable fractions [63, 64].

In addition, recent high-resolution imaging studies have demonstrated that Ni mobilisation by *O. chalcidica* can be highly localised at root tips, while bulk soil measurements may fail to capture such spatially confined processes [41]. Therefore, a longer experimental duration might have resulted in both a correlation in *w*_plant_(Ni) and *c*_DGT_(Ni) and an increase in *c*_DGT_ in planted vs. unplanted soils, reflecting the progressive mobilisation of Ni from the solid phase by *O. chalcidica*.

At the same time, the lack of apparent Ni mobilisation after 14 days is in accordance with the findings of a previous study applying the same (and additional) experimental soils as the present work [40]. There, differences in pore water Ni concentrations in pots planted with *O. chalcidica* vs. unplanted control pots were investigated for 7, 21, 49, and 71 days after transplantation. A significant increase in pore water Ni was first observed after 49 days. In addition to the limited experiment duration, the spatial scale of mobilisation processes may also have contributed to the lack of detectable changes in *c*_DGT_(Ni). A recent study using high-resolution DGT and planar optode imaging demonstrated that Ni mobilisation in the rhizosphere of *O. chalcidica* is highly localised at individual root tips [41].

**Fig. 6:**
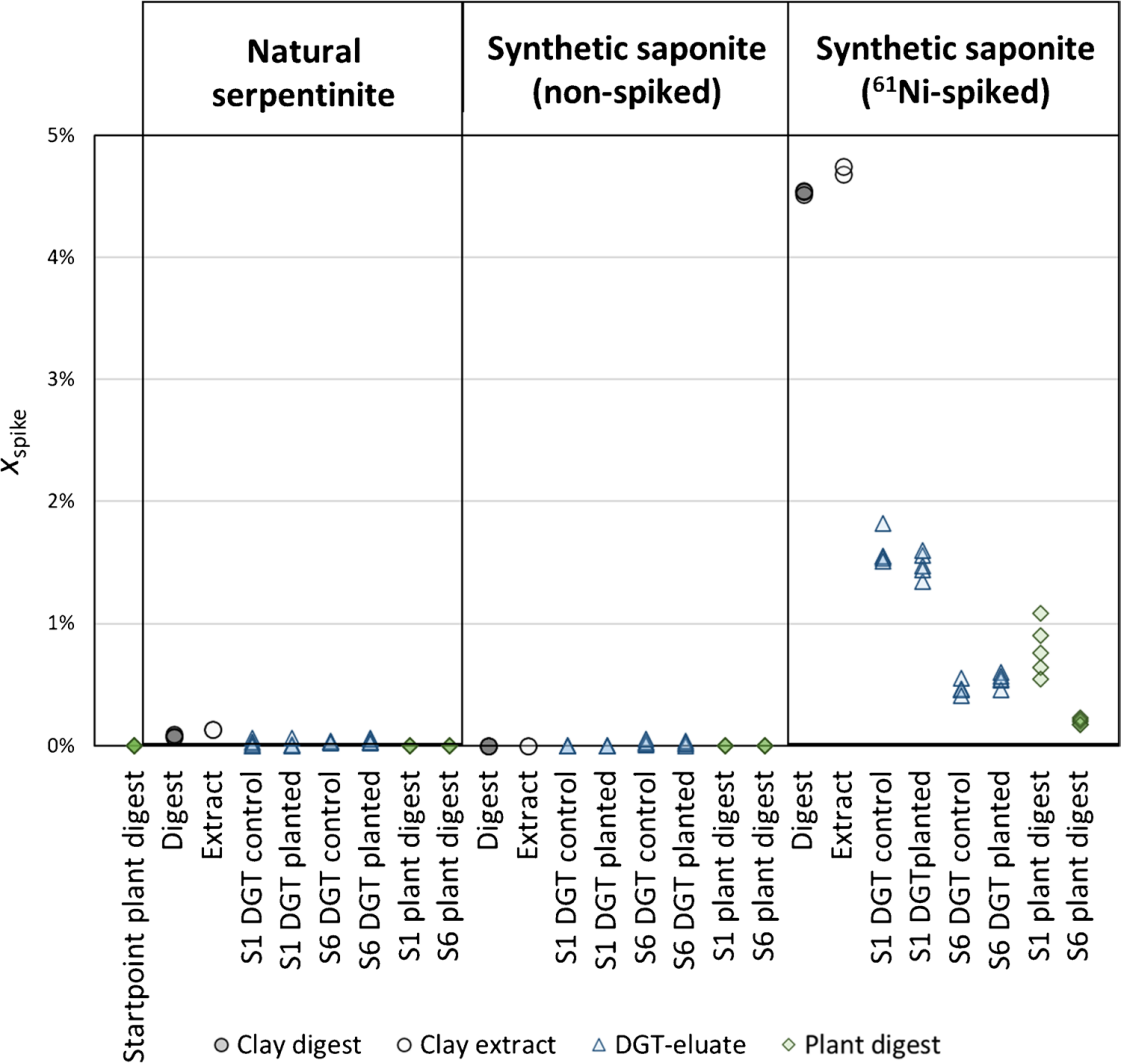
Molar fraction of the ^61^Ni spike (*x*_spike_) in the samples determined by IPD (startpoint plant digests, *n* = 10; other plant digests, *n* = 5; amendment digests, *n* = 3; amendment extracts, *n* = 2; DGT eluates, *n* = 5)

In contrast to the absence of apparent plant-induced Ni mobilisation based on the quantitative methods, the IPD approach revealed clear shifts in Ni isotope composition in both plant tissues and DGT-labile fractions, indicating uptake of the ^61^Ni tracer from the spiked amendment. Figure 6 illustrates the pathway of the tracer by showing *x*_spike_ in the individual groups of samples calculated through IPD.

As described in section ”RHIZOtest", the expected *x*_spike_ in the soil-amendment mixtures (calculated from pseudo-total Ni contents reported in literature [40]) would be 0.66% for S1 and 0.27% for S6. However, the DGT-labile fraction showed higher *x*_spike_ values, both in planted soils (1.5 ± 0.1% for S1 and 0.54 ± 0.05% for S6) and in unplanted control soils (1.6 ± 0.1% for S1 and 0.47 ± 0.05% for S6). This indicates that Ni originating from the amendment is proportionally overrepresented in the DGT-labile pool relative to its contribution to pseudo-total soil Ni.

For comparison, theoretical *x*_spike_ values calculated from DTPA-extractable Ni contents (derived from the same literature dataset as the pseudo-total contents [40]) would amount to 3.7% for S1 and 1.8% for S6. The lower *x*_spike_ observed in the DGT-labile fraction relative to the DTPA-based estimate suggests that the amendment-derived Ni is more strongly represented in the chelator-accessible pool than in the DGT-accessible fraction.

To assess whether *x*_spike_ differed significantly between the samples exposed to ^61^Ni-spiked versus non-spiked saponite, unpaired two-tailed *t*-tests assuming unequal variances (Welch’s *t*-tests) were performed. As expected, only the samples in contact with the spiked amendment exhibited a significant shift in Ni isotopic composition (*p* < 0.01).

The extent of tracer uptake in *O. chalcidica* varied by soil type. In S1, a stronger shift towards the tracer was observed. Because of the lower pseudo-total and DTPA-extractable Ni levels in S1, *x*_amendment_ is significantly higher in plant samples grown on S1 compared to S6 (*p* = 0.00486 based on a Welch’s *t*-test), as shown in Table 3. This indicates that a larger proportion of Ni taken up by the plants grown on S1 originated from the amendment, which points towards increased Ni mobilisation from the amendment by the plants grown on the soil with lower Ni contents.

**Table 3:** Estimated molar fraction of spike (*x*_spike_) and amendment (*x*_amendment_) in the plant samples grown on soil with spiked amendment, and corresponding mass fractions of Ni stemming from the spike and the amendment as a whole. The given uncertainty represents standard deviation between replicates (*n* = 5 per group)

Soil	*x*_spike_/%	*x*_amendment_/%	*w* (Ni)/ng g^−1^		
			Total	From spike	From amendment
S1	0.87 ± 0.23	19.3 ± 5.0	35,000 ± 19,000	310 ± 154	6700 ± 3400
S6	0.350 ± 0.081	7.7 ± 1.8	33,000 ± 22,000	110 ± 53	2400 ± 1100

While total Ni mass fractions in plant shoots were comparable between soils (35,000 ± 19,000 ng g^−1^ for S1 and 33,000 ± 22,000 ng g^−1^ for S6), the contribution of the amendment-derived Ni was significantly higher in S1. On average, plants grown on S1 contained almost three times more Ni originating from the amendment (6700 ± 3400 ng g^−1^) than those grown on S6 (2400 ± 1100 ng g^−1^).

This indicates that the synthetic material contributed more strongly to the plant-available Ni pool in the lower Ni soil. Whether this is due to a greater release of Ni from the amendment into the labile pool (independent of plant activity) or to an increased uptake by the plant under Ni-limited conditions remains to be investigated. One possible explanation is that *O. chalcidica* adjusts its uptake strategy depending on the background Ni availability, which might involve the activation of different physiological mechanisms [41].

Importantly, our findings demonstrate that the observed differences would not have been detectable through total quantitative Ni measurements alone. The tracer-based approach thus provides a powerful tool to distinguish between Ni derived from background soil pools and Ni mobilised from targeted amendments, and thereby enables a more detailed understanding of Ni uptake dynamics.

## Conclusion

In this work, a ^61^Ni-spiked saponite was successfully synthesised and employed in a 14-day RHIZOtest experiment with the Ni hyperaccumulator plant *O. chalcidica*. The presented methodology supports research on sustainable soil remediation by delivering a highly sensitive analytical tool for tracing plant Ni uptake pathways using a ^61^Ni spike.

This study represents a novel application of stable isotope spiking with ^61^Ni in combination with synthetic saponite, providing a controlled and scalable method for studying Ni mobilisation and uptake by hyperaccumulator plants. Despite the absence of measurable plant-induced Ni mobilisation at the bulk soil scale, IPD revealed that the isotopic tracer was taken up by *O. chalcidica*, particularly in the soil with lower Ni mass fractions. This suggests that *O. chalcidica* mobilises Ni from the synthetic clay phase, even when the bioavailable pool appears unaffected in bulk measurements.

The contrast between the sensitivity of the isotope-based approach and the quantitative approach (observable differences in *c*_DGT_(Ni) in planted vs. unplanted soils) underlines the added value of stable isotope spiking. By enabling the detection of subtle plant uptake processes within short experimental time frames, the method provides valuable complementary insights into metal mobilisation, while also reducing experimental cost, duration, and complexity. By comparing the synthetic saponite amendment to natural serpentinite, the synthetic material was found not to negatively affect plant growth.

Together, the presented results demonstrate the potential of this approach for evaluating the environmental fate of metal-bearing materials. The ability to trace Ni uptake pathways with high sensitivity, even in the absence of detectable bulk mobilisation, can support mechanistic investigations of Ni mobilisation and uptake in phytomining and remediation contexts, and thereby aid the design of more effective phytomanagement strategies.

## Supplementary Information

Below is the link to the electronic supplementary material.

Supplementary file1 (PDF 389 KB)

Supplementary file2 (XLSX 292 KB)

## Data Availability

The authors declare that the data supporting the findings of this study are available within the paper and its Supplementary Information files. Should any raw data files be needed in another format, they are available from the corresponding author upon reasonable request.
