# Supplementary material for: Tracking nickel uptake pathways in hyperaccumulator plants using a 61Ni-enriched stable isotope tracer in soil
Source: Anal Bioanal Chem. 2026 May 2;418(14):4481–95. doi: 10.1007/s00216-026-06539-6 (PMC13375821; doi:10.1007/s00216-026-06539-6)
Supplement: Supplementary file 1 — Supplementary file1 (PDF 389 KB) [file 216_2026_6539_MOESM1_ESM.pdf]

Online Resource 1: Supplementary Information

Tracking nickel uptake pathways in hyperaccumulator plants using a ^{61}Ni-enriched stable isotope tracer in soil

Simone Trimmel[a], Alexander V. Epov[a], Nadine Abu Zahra[a], Tobias Berger[b], Thomas Prohaska[a], Markus Puschenreiter[c], Antonia Siebenbrunner[a], Alice Tognacchini[c], Stefan Wagner[a], Johanna Irrgeher[a]

[a]Montanuniversität Leoben, Department General, Analytical and Physical Chemistry, Chair of General and Analytical Chemistry, Leoben, Austria

[b]Montanuniversität Leoben, Department General, Analytical and Physical Chemistry, Chair of Physical Chemistry, Leoben, Austria

[c]BOKU University, Department of Forest- and Soil Sciences, Institute of Soil Research (IBF), Vienna, Austria

*Correspondence: johanna.irrgeher@unileoben.ac.at

ORCID:

Simone Trimmel:	0000-0002-2102-5392
Alexander Epov:	0009-0005-1008-5786
Nadine Abu Zahra:	0009-0008-2724-6752
Tobias Berger:	0009-0004-8581-7243
Thomas Prohaska:	0000-0001-9367-8141
Markus Puschenreiter:	0000-0002-7298-4163
Antonia Siebenbrunner:	0009-0004-9490-6686
Alice Tognacchini:	0000-0003-2156-7295
Stefan Wagner:	0000-0002-6880-8036
Johanna Irrgeher:	0000-0003-3192-0101

1. Reagents

For the saponite synthesis, iron chloride hexahydrate ($FeCl_3 \cdot 6\ H_2O$, Carl Roth GmbH, Germany), sodium hydroxide (NaOH, $w \geq 98$ %, p.a., Carl Roth GmbH, Germany), sodium silicate pentahydrate ($Na_2SiO_3 \cdot 5\ H_2O$, VWR, Belgium), aluminium chloride ($AlCl_3$, anhydrous, VWR Rectapur, USA), magnesium chloride hexahydrate ($MgCl_2 \cdot 6\ H_2O$, $w \geq 99$ %, p.a., Carl Roth GmbH, Germany), urea (CH_4N_2O, high purity grade, VWR, USA), L-histidine ($C_6H_9N_3O_2$, VWR, USA) and HCl (p.a. grade, Chem Lab, Belgium) were applied.

Boric acid (H_3BO_3, Merck, Germany), potassium dihydrogen phosphate (KH_2PO_4, ≥ 99 %, Sigma-Aldrich, USA), calcium nitrate tetrahydrate ($Ca(NO_3)_2 \cdot 4\ H_2O$, ≥ 98 %, Carl Roth GmbH, Germany), magnesium sulphate heptahydrate ($MgSO_4 \cdot 7\ H_2O$, ≥ 99 %, Carl Roth GmbH, Germany), copper sulphate pentahydrate ($CuSO_4 \cdot 5\ H_2O$, p.a., Merck, Germany), manganese chloride tetrahydrate ($MnCl_2 \cdot 4\ H_2O$, ≥ 98 %, Sigma-Aldrich, USA), zinc sulphate heptahydrate ($ZnSO_4 \cdot 7\ H_2O$, ≥ 97 %, Carl Roth GmbH, Germany), sodium molybdate dihydrate ($Na_2MoO_4 \cdot 2\ H_2O$, ≥ 99 %, Sigma-Aldrich, USA), sodium ferric ethylenediaminetetraacetate (NaFe(III)EDTA, ≥ 98 %, Carl Roth GmbH, Germany), and potassium nitrate (KNO_3, ≥ 99 %, Carl Roth GmbH, Germany) were used to prepare the nutrient solution for the rhizotests.

Chelex 100 resin for diffusive gradients in thin films (DGT) was purchased from Sigma-Aldrich, USA.

2. MVI calibration standards

The ICP multi-element calibration standard stock solution VI (MVI, Merck Certipur, Germany) contains 989 mg L^{-1} of calcium (Ca); 102 mg L^{-1} of beryllium (Be); 100 mg L^{-1} of iron (Fe); 99 mg L^{-1} of zinc (Zn); 10 mg L^{-1} of silver (Ag) and strontium (Sr); 9.9 mg L^{-1} of cadmium (Cd), cobalt (Co), chromium (Cr), copper (Cu), gallium (Ga), lithium (Li), manganese (Mn), molybdenum (Mo), Ni, rubidium (Rb), tellurium (Te) and thallium (Tl); 9.8 mg L^{-1} of aluminium (Al), bismuth (Bi), magnesium (Mg), lead (Pb), uranium (U) and vanadium (V) and 9.7 mg L^{-1} of barium (Ba) and sodium (Na).

The stock solution was diluted with nitric acid (HNO_3, $w = 2$ %) to obtain a 15-point series of calibration standards ranging from (nominal) 0.005 to 150 ng g^{-1} lithium (Li). The specification of traceability of the respective elements contained in the MVI solution can be found in **Table S1**. For the other standard solutions, no statement regarding traceability is given.

Table S1. Specification of traceability for the elements contained in ICP multi-element solution VI (Merck Certipur).

Element	NIST Standard Reference Material
**Ag**	SRM 3151
**Al**	SRM 3101a
**As**	SRM 3103a
**B**	SRM 3107
**Ba**	SRM 3104a
**Be**	SRM 3105a
**Bi**	SRM 3106
**Ca**	SRM 3109a
**Cd**	SRM 3108
**Co**	SRM 3113
**Cr**	SRM 3112a
**Cu**	SRM 3114
**Fe**	SRM 3126a
**Ga**	SRM 3119a
**K**	SRM 3141a
**Li**	SRM 3129a
**Mg**	SRM 3131a
**Mn**	SRM 3132
**Mo**	SRM 3134
**Na**	SRM 3152a
**Ni**	SRM 3136
**Pb**	SRM 3128
**Rb**	SRM 3145a
**Se**	SRM 3149
**Sr**	SRM 3153a
**Te**	SRM 3156
**Tl**	SRM 3158
**U**	SRM 3164
**V**	SRM 3165
**Zn**	SRM 3168a

3. Total mineral analysis of natural serpentinite

After collection, the serpentinite rock was dried and ground in a vibrating disc mill to analytical fineness using an agate grinding jar to preserve low blank levels for geochemical trace analysis. Preparation for X-ray diffraction

(XRD) analysis was done using the backloading method. The measurement was carried out in an XPert Pro MPD diffractometer (Malvern Panalytical, UK) with automatic divergence slit, Cu LFF tube, 45 kV, 40 mA and an X'Celerator detector. The measurement time was 25 s, with an increment of 0.017° from 3° to 70° 2 theta. The qualitative mineral composition was determined from the obtained images. The results were further refined with the Software X'Pert HighScore Plus (Malvern Panalytical, UK) using the Rietveld method. From this, the semi-quantitative mineral content given in **Table S2** was calculated.

Table S2. Semi-quantitative composition of the sampled natural serpentinite rock.

Mineral	Serpentine	Chlorite	Magnetite	Magnesite
w / %	85	13	1-2	traces

As expected, the rock sample contains lizardite as the main component. Lizardite is the most common representative from the serpentine mineral group and is characterised by a massive, fine-grained structure. As a second mineral group, chlorite could be determined in smaller proportions. Small amounts of magnesite were detectable. Magnesite, a typical weathering mineral of serpentinites could be detected in traces. Discrete nickel minerals are below the limit of detection (x_D).

4. Saponite synthesis

Ni powder, either of natural isotopic composition (Merck, Germany) or ^{61}Ni-enriched (Trace Sciences International, Canada) was dissolved in boiling p.a. grade HCl (w = 37 %). The solution was then dried at 100 °C to obtain anhydrous nickel chloride ($NiCl_2$). All other reagents used for the synthesis were prepared using ultra-pure water, where applicable. Two separate batches of the synthesis were conducted for each material (^{61}Ni-enriched and natural Ni powder), using four separate round-bottomed flasks. For each flask, 4.2 mL NaOH (c = 5 mol/L) were vortexed with 4.2 mL $AlCl_3$ (c = 1 mol/L) until a transparent solution was obtained. The mixture was added dropwise to a round-bottomed flask containing 20 mL Na_2SiO_3 (c = 1.2 mol/L) under vigorous magnetic stirring. The flasks were heated under reflux cooling and continuous magnetic stirring to 105 °C in an oil bath.

For each flask, 17 mL urea (c = 5 mol/L), 12 mL $MgCl_2$ (c = 1.2 mol/L), 3-3.5 mL $NiCl_2$ (c = 1.2 mol/L), 5-5.5 mL $FeCl_3$ (c = 0.5 mol/L), 17.5 mL L-histidine · HCl (c = 0.4 mol/L) and 18 mL HCl (p.a., c = 2 mol/L) were combined in a beaker and topped off to 100 mL with ultra-pure water. The flasks were then heated at 105 °C for one hour, they were cooled in a water bath for 1-2 minutes and their contents were diluted to 50 mL with ultra-pure water. The mixtures prepared in the beakers were added to the flasks and the pH was adjusted to 6.8-7.4. Without stirring, the flasks were heated to 105 °C for one week in an oil batch.

After the synthesis was completed, the mixtures were collected in 50 mL polyethylene (PE) tubes and centrifuged for 5 minutes at 5000 rpm. The solution was decanted and the remaining saponite was redispersed in ethylenediaminetetraacetic acid (EDTA, c = 0.05 mol/), followed by overhead shaking at 20 rpm for one hour before centrifuging and decanting again. This washing step was repeated 15 times to remove the bioavailable

fraction of Ni as thoroughly as possible. At each step, the supernatant was collected for multielement and Ni isotopic characterisation. The last step was followed by washing with ultra-pure H_2O and air-drying. The air-dried saponite from the individual flasks was thoroughly homogenised using mortar and pestle. Natural serpentinite was washed, dried and homogenised in an analogue way for the control group.

4. Multielement analysis (NexION 5000)

The instrumental parameters for the multielement measurements using the NexION 5000 ICP-MS/MS instrument (PerkinElmer, USA) are given in **Table S3**.

Table S3. Instrumental parameters for the ICP-MS/MS multielement analysis using the NexION 5000 (PerkinElmer, USA) in standard and dynamic reaction cell (DRC) mode.

**Parameter**	**Setup**	
**Mode**	Standard	DRC
m/z	7, 9, 13, 23, 24, 25, 27, 31, 35, 42, 43, 44, 45, 46, 48, 54, 55, 57, 58, 59, 60, 61, 62, 63, 64, 65, 66, 67, 68, 70, 111, 112, 114, 116, 121, 122, 123, 124, 206, 207, 208	39, 41, 56
**Cell gas**	None	Ammonia (NH_3)
**Cell gas flow**	-	0.7 mL min^{-1}
**Spray chamber temperature**	5 °C	
**Interface cones**	Ni	
**Nebulizer**	PFA-ST-40 44296	
**Nebulizer gas flow**	0.97-0.99 mL min^{-1}	
**RF power**	1600 W	
**Plasma gas flow**	16 L min^{-1}	
**Auxiliary gas flow**	1.2 L min^{-1}	
**Data acquisition mode**	6 sweeps/reading, 1 reading/replicate, 6 replicates	
**Dwell time per replicate**	25-150 ms	
**Integration time**	150-900 ms	
**RPa**	0-0.015 V	0 V
**RPq**	0.25 V	0.45 V
**Total time/sample**	2 min 14 s	

The elemental mass fractions in the samples which were used for data evaluation and interpretation, and which are comprehensively presented in Online Resource 2, **Table B1**, were obtained from measuring the samples on the mass-to-charge (*m*/*z*) ratios listed in **Table S4**.

Table S4. Measurement modes and *m*/*z* ratios which were selected to quantify the analytes in the samples

Analyte	*m/z*	Mode
**Li**	7	STD
**Be**	9	STD
**Na**	23	STD
**Mg**	25	STD
**Al**	27	STD
**P**	31	STD
**K**	41	NH_3 DRC
**Ca**	43	STD
**Mn**	55	STD
**Fe**	56	NH_3 DRC
**Co**	59	STD
**Ni**	60	STD
**Cu**	65	STD
**Zn**	64	STD
**Cd**	114	STD
**Pb**	207	STD

5. Ni isotope ratio analysis (NexION 2000)

The instrumental parameters for the Ni isotope ratio measurements using the NexION 2000 ICP-MS instrument (PerkinElmer, USA) are given in **Table S5**.

Table S5. Instrumental parameters for the ICP-MS Ni isotope ratio analysis using the NexION 2000 (PerkinElmer, USA).

**Parameter**	**Setup**
**Mode**	Standard
m/z	58, 60, 61, 62, 64, 66, 68, 115
**Cell gas**	None
**Cell gas flow**	-
**Spray chamber temperature**	5 °C
**Interface cones**	Ni
**Nebulizer**	PFA-ST-40 44296
**Nebulizer gas flow**	0.97-0.99 mL min^{-1}
**RF power**	1600 W
**Plasma gas flow**	16 L min^{-1}
**Auxiliary gas flow**	1.2 L min^{-1}
**Data acquisition mode**	1 sweep/reading, 1 reading/replicate, 900 replicates
**Dwell time per replicate**	20-50 ms
**Integration time**	20-50 ms
**RPa**	0 V
**RPq**	0.25 V
**Total time/sample**	2 min 52 s

6. DGT data processing

The equations (eq.) applied in DGT data processing are based on established literature (1).

The mass of analyte which accumulated on the DGT binding layer was calculated according to eq. S1.

$$M = \frac{c_\mathrm{e}(V^\mathrm{bl} + V_\mathrm{e})}{f_\mathrm{e}} \qquad \text{eq. S1}$$

M: mass of analyte accumulated on binding layer (ng)

c_e: measured concentration in the eluent (ng/mL)

V^bl: volume of the binding layer

V_e: volume of the eluent

f_e: elution efficiency

Thereby, V^bl was obtained according to eq. S2.

$$V^{\mathrm{bl}} = {r^{\mathrm{bl}}}^2 \cdot \pi \cdot \delta^{\mathrm{r}} = 2\ \mathrm{cm}^3 \qquad \text{eq. S2}$$

r^{bl}: disc radius (this study: 1.25 cm)

δ^{r}: thickness of binding layer (this study: 0.04 cm)

The diffusion coefficient of the analyte in the material diffusion layer D^{mdl} is obtained for the experimental temperature (this study: T = 21.3 °C) based on the diffusion coefficient at 25 °C (D^{mdl}_{25} = 5.77E-06 cm^2 s^{-1} (2)) by rearranging the Stokes-Einstein equation (3) as shown in eq. S3.

$$D^{\mathrm{mdl}} = 10^{\frac{1.37023(T-25)+0.000836(T-25)^2}{109+T}+\lg\frac{D^{\mathrm{mdl}}_{25}(273+T)}{298}} = 5.21\text{E-06 cm}^2\ \text{s}^{-1} \qquad \text{eq. S3}$$

7. Plant biomass

To further investigate potential drivers of the observed variation in plant biomass, correlation analyses were conducted between biomass and the mass fractions of the analytes in plant tissues. Given the differing growth durations of startpoint (3 weeks) and non-startpoint (5 weeks) samples, and one being exposed to soil and one not, correlations were evaluated both across all plant samples and for the two groups separately.

Across all plant samples (n = 40), significant negative correlations were observed between biomass and the mass fractions of several nutrients. The Pearson correlation coefficients (r) and corresponding p-values for the most notable observations (Mg, K, Ca, Fe, Cu and Zn) are shown in **Table S6**. This trend is consistent with the well-documented decrease in nutrient contents in plants with higher biomass due to increased distribution volume, resulting in dilution of the nutrients across the plant body (4).

Table S6. Pearson coefficients r for the correlations between plant biomass and selected elemental mass fractions. Statistically significant observations ($p < 0.05$) are highlighted with bold letters.

Group	Parameter	Mg	K	Ca	Fe	Cu	Zn
**All plant samples**	r	**-0.57**	**-0.72**	**-0.76**	**-0.48**	**-0.68**	**-0.63**
	p	**< 0.01**	**< 0.01**	**< 0.01**	**0.01**	**< 0.01**	**< 0.01**
**Non-startpoint only**	r	**-0.37**	**-0.51**	**-0.52**	**-0.53**	**-0.55**	**-0.61**
	p	**0.05**	**0.01**	**< 0.01**	**0.01**	**0.01**	**< 0.01**
**Startpoint only**	r	-0.32	-0.59	**-0.70**	-0.40	-0.49	-0.40
	p	0.36	0.07	**0.02**	0.33	0.15	0.25

References

1. Davison W, Zhang H. Introduction to DGT. In: Davison W, editor. Diffusive Gradients in Thin-Films for Environmental Measurements. Cambridge: Cambridge University Press; 2016. p. 3-4.
2. DGT Research. Diffusion Coefficients 2025 [cited 2025 26th of June]. Available from: https://www.dgtresearch.com/diffusion-coefficients/.
3. Atkins P, de Paula J. Atkins' Physical Chemistry. 8th edition ed. Oxford: Oxford University Press; 2006.
4. Jarrell WM, Beverly RB. The Dilution Effect in Plant Nutrition Studies. In: Brady NC, editor. Advances in Agronomy. 34: Academic Press; 1981. p. 197-224.
